# The Influence of Monosaccharide Composition on the Bioactivity of Medicinal Plant Polysaccharides

**DOI:** 10.3390/ijms27073075

**Published:** 2026-03-27

**Authors:** Xinhui Fan, Ke Li, Maohui Yang, Xuemei Qin, Zhenyu Li, Yuguang Du

**Affiliations:** 1 Modern Research Center for Traditional Chinese Medicine, Shanxi University, Taiyuan 030006, China; xhfan@nenu.edu.cn (X.F.);; 2 The Key Laboratory of Chemical Biology and Molecular Engineering of Ministry of Education, Shanxi University, Taiyuan 030006, China; 3 Engineering Research Center of Glycoconjugates, School of Life Sciences, Ministry of Education, Northeast Normal University, Changchun 130024, China; 4 State Key Laboratory of Biochemical Engineering, Institute of Process Engineering, Chinese Academy of Sciences, Beijing 100700, China; 5 Institute of Chinese Materia Medica, China Academy of Chinese Medical Sciences, Beijing 100700, China

**Keywords:** bioactivity, medicinal plant polysaccharides, monosaccharide composition

## Abstract

Polysaccharides are natural polymers that are widely found in medicinal plants. Structurally, they are complex molecules composed of long chains of monosaccharide units linked by glycosidic bonds. Modern pharmacological research shows that the bioactivity of polysaccharides is closely related to their monosaccharide composition. This review summarises the monosaccharide composition of 210 polysaccharides from 72 medicinal plants. They were classified into 10 types through principal component analysis (glucans; homogalacturonan; galactans; arabinogalactans; mannans; glucomannans; arabinans; xylans; fructans; rhamnogalacturonan-I). The relationship between monosaccharide composition and biological activity was further analysed. The results are as follows: glucans make significant contributions to immunomodulation, antioxidant activity, and gut microbiota regulation; galactans are crucial for antioxidant effects, immunomodulation, and gut microbiota regulation; mannans play a key role in immunomodulation, antitumor activity, and neuroprotection; fructans are vital for gut microbiota regulation, immunomodulation, and antioxidant effects; and pectins exhibit notable immunomodulatory, antioxidant, and hypoglycaemic properties. Consequently, developing polysaccharides from medicinal plant resources based on their monosaccharide composition is expected to speed up the search for polysaccharides with high biological activity and provide a theoretical reference for polysaccharide research.

## 1. Introduction

Polysaccharides are natural macromolecule polymers of long chains of monosaccharide units linked via glycosidic bonds and are widely found in medicinal plants [1]. Due to their non-toxicity and abundant availability, they have garnered increasing research attention in recent years [2].

Modern pharmacological studies have shown that medicinal plant polysaccharides have a variety of biological activities, including immune regulation, protection of the liver, protection of nerves, anti-oxidation, anti-fatigue, regulation of intestinal flora, and regulation of blood glucose [3].

Polysaccharides possess highly complex structural characteristics, including molecular weight, monosaccharide composition, glycosidic bond configuration, and functional groups [4]. These structural features determine and influence the biological activity of polysaccharides. Among these, monosaccharides constitute the most fundamental units of the primary structure of polysaccharides and form the basis for other advanced structures [5]. They not only influence the physicochemical properties of polysaccharides, such as functional group, electrification, chain length, and spatial conformation [6], but are also among the most easily detectable indicators. For example, Feng et al. [7] found that an increase in uronic acid content induced by ultrasonic treatment enhanced the foam capacity, thermal stability, antioxidant activity, and antitumor activity of polysaccharides from *Sagittaria sagittifolia*. Yu et al. [8] highlighted the composition and proportion of monosaccharides as key structural features influencing the probiotic activity of polysaccharides. They noted that neutral sugars in the side chains of sugar beet pulp polysaccharides are more readily fermented by gut microbiota, and that intestinal flora preferentially utilise arabinose, glucose, fucose, and galacturonic acid, in that sequence.

Although various biological activities of medicinal plant polysaccharides have been extensively studied, a review focusing on how monosaccharide composition influences their biological functions remains relatively scarce. An analysis of the relationship between monosaccharide composition and specific biological activities is crucial for understanding the mechanisms of action of polysaccharides.

However, this field of research faces a fundamental challenge: most published bioactivity data currently originate from incompletely isolated, highly heterogeneous mixed polysaccharide fractions. Directly exploring the correlation between monosaccharide composition and activity based on such data is prone to introducing bias, thereby reducing the reliability of conclusions. To address this issue, this study screened the literature by searching PubMed and Web of Science databases using keywords such as “polysaccharides” and “Latin names of 72 medicinal plants”. All the selected publications underwent a rigorous evaluation to ensure direct relevance to this review. To enhance the reliability of structure–activity relationship analysis, only studies involving highly homogeneous polysaccharide fractions-systematically isolated, purified, and structurally characterised were included. Additionally, publications were required to explicitly provide monosaccharide composition data and corresponding bioactivity assay results. This screening strategy established a relatively reliable and comparable data foundation for this research.

This review summarises high-quality literature from recent years concerning the influence of monosaccharide composition on the biological activity of polysaccharides. It is hoped that this will provide new perspectives for understanding the relationship between the structure and function of medicinal plant polysaccharides.

## 2. Main Monosaccharides Description

**Figure 1:**
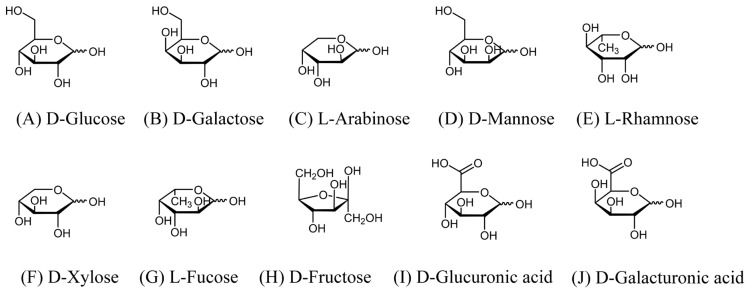
Main types of monosaccharides composing medicinal plant polysaccharides.

The primary monosaccharide types in medicinal plant polysaccharides include glucose, galactose, arabinose, mannose, rhamnose, xylose, fucose, glucuronic acid, galacturonic acid, and fructose. The monosaccharides are classified as pentoses and hexoses based on the number of carbon atoms [9]. Xylose and arabinose are classified as pentoses. Meanwhile, glucose, galactose, mannose, rhamnose, fucose, glucuronic acid, galacturonic acid, and fructose are classified as hexoses or their derivatives. Furthermore, uronic acids are a class of monosaccharide derivatives characterised by the structural oxidation of the hydroxyl group (typically at C6) to a carboxyl group (-COOH) [10]. The types of uronic acid that make up medicinal plant polysaccharides mainly include glucuronic acid and galacturonic acid. Their structures are shown in Figure 1.

## 3. Classification of Medicinal Plant Polysaccharides

**Figure 2:**
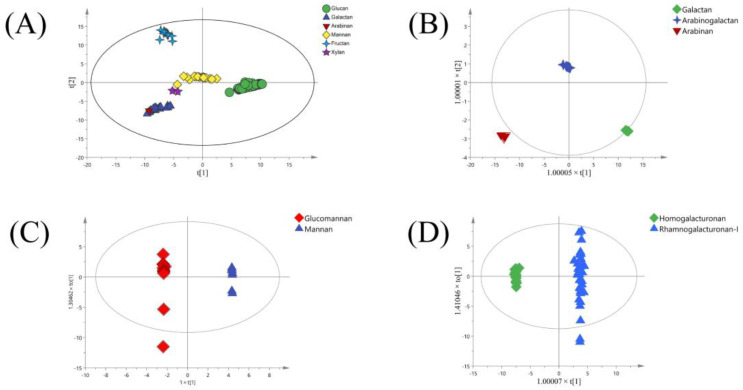
PCA scatter plots of monosaccharide composition of medicinal plant polysaccharides. (**A**): neutral polysaccharides (glucans, galactans, arabinans, mannans, fructans, and xylans); (**B**): galactans (galactans, arabinogalactan, arabinans); (**C**): mannans (mannans, glucomannans); (**D**): acidic polysaccharides (homogalacturonan, rhamnogalacturonan-I).

**Figure 3:**
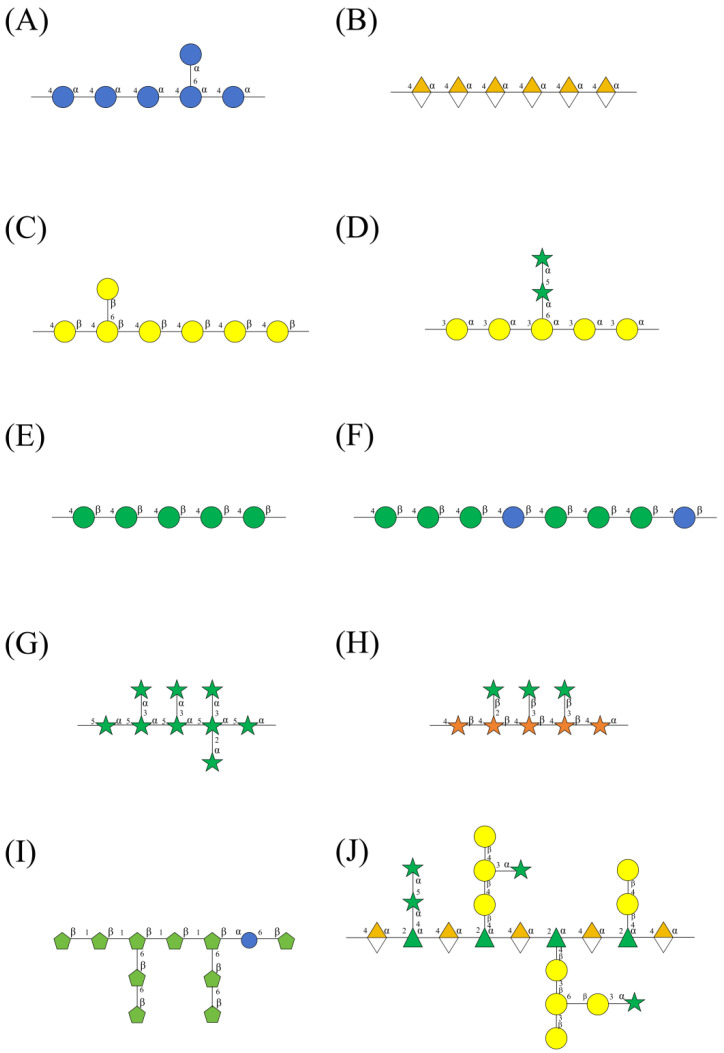
The hypothetical chemical structure of 10 polysaccharides. (**A**): glucans; (**B**): homogalacturonan; (**C**): galactans; (**D**): arabinogalactans; (**E**): mannans; (**F**): glucomannan; (**G**): arabinans; (**H**): xylans; (**I**): fructans; (**J**): rhamnogalacturonan-I. All shapes and colors comply with the Symbol Nomenclature for Glycans guidelines (https://www.ncbi.nlm.nih.gov/glycans, accessed on 4 February 2026).

This article summarises the monosaccharide composition of 210 polysaccharides from 72 medicinal plants. These are listed in Table 1 and Figure 2. Polysaccharides in medicinal plants may be classified as neutral polysaccharides or acidic polysaccharides based on whether their monosaccharide composition includes uronic acids. Neutral polysaccharides are primarily classified into six categories: glucans, galactans, arabinans, mannans, fructans, and xylans(Figure 2A). In the classification of galactans, arabinogalactan constitutes the primary component, whereas the proportion of pure galactans (gal > 90%) is comparatively low (Figure 2B). In the classification of mannans, glucomannan constitutes the primary component, whereas the proportion of pure mannans (man > 90%) is comparatively low (Figure 2C). Acidic polysaccharides are primarily pectins, which are categorised into two main types (Figure 2D) based on their monosaccharide composition: homogalacturonan(HG) and rhamnogalacturonan-I(RG I). Their structures of 10 polysaccharides are shown in Figure 3.

## 4. The Correlation Between Activities and Monosaccharide Composition of Medicinal Plant Polysaccharides

**Figure 4:**
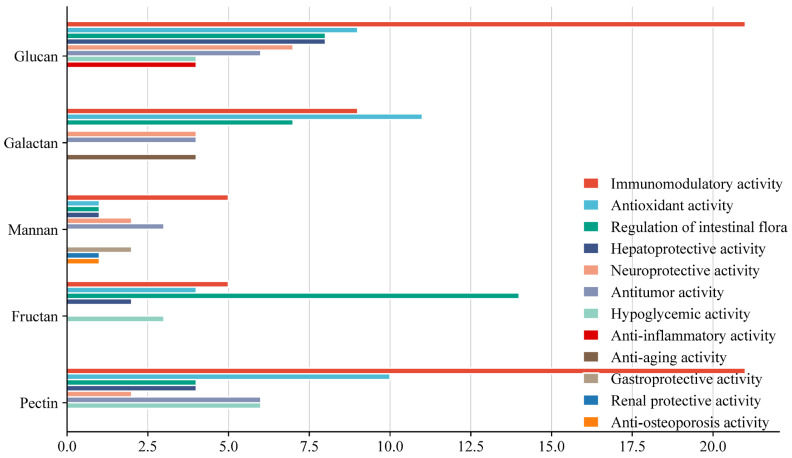
The Correlation Between Activities and Monosaccharide Composition of Medicinal Plant Polysaccharides.

Based on the analysis of the collected literature in Table 1, it appears that there may be some relationship between monosaccharide composition and the biological activity of medicinal plant polysaccharides (Figure 4 and Table 2). For each polysaccharide, we select the top three bioactivities with the highest number of literature reports for discussion.

Glucans play a critical role in immune regulation [13], antioxidant effects [47], and the regulation of gut microbiota [30]. Galactans play a critical role in antioxidant effects [94], immune regulation [79], and the regulation of gut microbiota [84]. Mannans play a critical role in immune regulation [124], antitumor effects [128], and neuroprotection [130]. Fructans play a critical role in the regulation of gut microbiota [143], immune regulation [154], and antioxidant effects [160]. Pectins play a critical role in immune regulation [171], antioxidant effects [190], and lowering blood sugar [207]. Furthermore, due to the limited variety of arabinan and arabinoxylan found in medicinal plants, they are not discussed in this article.

## 5. Effects of the Monosaccharide Composition on the Bioactivity of Medicinal Plant Polysaccharides

### 5.1. Immunomodulatory Activity

Medicinal plant polysaccharides are important macromolecules that can strongly affect the immune system and have the potential to be used as immunomodulators with broad clinical applications [221]. Table 2 reveals that glucans and pectins exhibit excellent immunomodulatory activity.

The monosaccharide composition of glucans is simple, with glucose content exceeding 90%. For instance, a glucan (glucose content: 89.0%) isolated from Astragalus membranaceus promotes the polarisation of M0-type macrophages to the M1-type and repolarises M2-type to M1-type via the TLR4-MyD88-NF-κB signalling pathway [11]. Another glucan from the same plant (glucose content: 95.7%) significantly alleviates the immunosuppression in mice by enhancing immune organ indices, stimulating immune cell proliferation, and reducing intestinal inflammation, mechanisms linked to activation of TLR4 and MAPK signalling [12]. A further glucan (91.6% glucose) isolated from Astragalus membranaceus suppresses pro-inflammatory cytokines while elevating anti-inflammatory mediators through coordinated regulation of the SIRT1/PGC-1α/NF-κB and FXR-mediated pathways [14]. Similarly, a glucan (glucose content: 97.5%) from Angelica dahurica enhances phagocytosis and promotes the release of NO, TNF-α, and IL-6 from RAW264.7 cells, accompanied by up-regulation of iNOS, TNF-α, and IL-6 mRNA and increased phosphorylation of p65, p38, ERK, and JNK proteins [20]. Glucans from Angelica sinensis also exhibit immunostimulatory effects: one (97.8% glucose) could cause the proliferation of the lymphocyte, upregulate and stimulate the production of IFN-γ, IL-2, IL-6, and TNF-α secretion, and increase the ratio of CD3(+)CD56(+) cells to some extent [21]; another (95.7% glucose) promotes surface molecule expression on RAW264.7 cells, stimulates T and B lymphocytes proliferation and cytokine secretion, and improves immune organ indices, cytokine levels, and T lymphocyte subtype in cyclophosphamide-induced immunosuppressed mice [27].

In contrast, the monosaccharide composition of pectin is highly complex, but galacturonic acid is the key component that distinguishes it from other types of polysaccharides. A pectin from Panax ginseng (49.3% galacturonic acid) promotes TLR2, NF-κB, and TRAF6 protein expression levels, thereby enhancing macrophage phagocytosis, splenic lymphocyte proliferation, and secretion of NO, IL-1β, IL-6, and TNF-α [171]. Another pectin (47.7% galacturonic acid) from Panax ginseng increases IgG, IgG1, and IgG2a production, elevates the splenocyte proliferation index, and promotes expression of GATA-3, T-bet, IFN-γ, and IL-4 in H1N1 vaccine-immunised mice, mediated through the activation of the TLR4-dependent pathway via up-regulation of TLR4, MyD88, TRAF-6, and NF-κB proteins and genes [175]. A pectin (17.1% galacturonic acid) from Panax notoginseng triggers the DC-induced T-cell immune response, as indicated by the higher expressions of CD4, CD8, CD69, and MHC II in T cells with increased secretion of INF-β. Furthermore, it could bind to the pattern recognition receptors (PRR) of Toll-like receptor 4 (TLR 4), Toll-like receptor 2 (TLR 2), mannose receptor (MR), and activate TLR4/TLR2-NF-κB signaling pathway [180].

**Figure 5:**
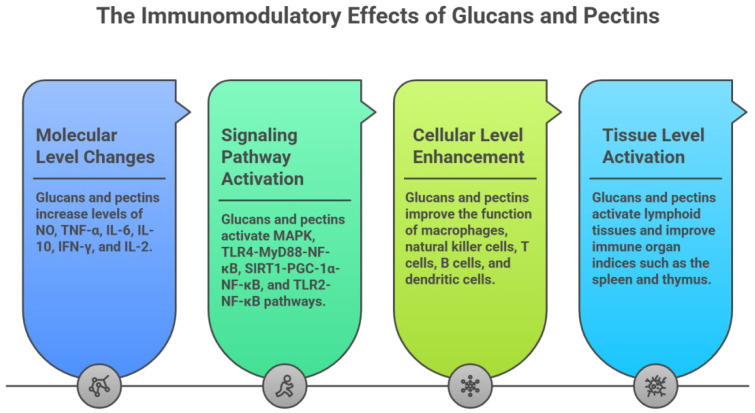
Immunomodulatory Mechanisms of Medicinal Plant Polysaccharides. Glucans and pectins modulate systemic immune homeostasis by acting on immune organs (spleen and thymus), cells (macrophages, natural killer cells, T-lymphocyte (T cells), B-lymphocyte (B cells), and dendritic cells), and cytokines (Nitric Oxide (NO), Tumor Necrosis Factor-α (TNF-α), Interleukin-6 (IL-6), Interleukin-10 (IL-10), Interferon-γ (IFN-γ), and Interleukin-2 (IL-2)). The signaling pathways involved in this process include mitogen-activated protein kinase pathway (MAPK), toll-like receptor 4-myeloid differentiationfactor 88-nuclear factor-κB pathway (TLR4-MyD88-NF-κB), sirtuin 1-peroxisome proliferator-activated receptor-γ coactivator 1α-nuclear factor-κB pathway (SIRT1-PGC-1α-NF-κB), and toll-like receptor 2-nuclear factor-κB pathway (TLR2-NF-κB).

In summary, both glucans and pectins modulate systemic immune homeostasis by acting on immune organs, cells, and cytokines. Their consistent mechanisms of action are illustrated in Figure 5. Additionally, due to structural differences, they all possess characteristic immune receptors (glucans: Dectin-1, CR3, and pectins: MR, Gal-3) [180]. These features support their further development as potential immunomodulatory agents.

### 5.2. Antioxidant Activity

Oxidative stress is caused by a variety of oxygen-derived free radicals (ROS), such as superoxide anion, hydrogen peroxide, and hydroxyl radical. Elevated ROS levels can damage proteins, lipids, and DNA, ultimately leading to cell death [222]. Due to their notable antioxidant properties, polysaccharides have attracted growing research interest in recent years. As shown in Table 2, glucans, galactans, and pectins all demonstrate considerable antioxidant activity.

A glucan from Dendrobium officinale (77.8% glucose) significantly alleviates glial cell activation, enhances antioxidant enzyme activities, and decreases malondialdehyde (MDA) content in senescent mice [45]. A glucan (glucose content: 100%) from Polygonum multiflorum exhibits strong activity against free radicals, lipid oxidation, and protein glycation, with IC_50_ values of 0.47, 0.6, and 0.93 mg/mL for scavenging superoxide anion, hydroxyl radical (OH), and hydrogen peroxide (H_2_O_2_), respectively [47]. Another glucan from Pouteria campechiana (86.6% glucose) shows potent scavenging capacity toward 1,1-diphenyl-2-picrylhydrazyl (DPPH), 2,2′-azino-bis-(3-ethylbenzthiazoline-6-sulphonic acid) (ABTS), OH, and superoxide radicals [50]. Similarly, a glucan from Fallopia multiflora (100% glucose) displays high hydroxyl radical scavenging activity and reducing capacity in vivo, increasing serum superoxide dismutase (SOD) and glutathione peroxidase (GSH-Px) activities while lowering MDA levels [53].

In the classification of galactans, arabinogalactan constitutes the primary component. Galactose serves as the primary backbone and framework, while arabinose exists as a side-chain modification. The combined content of galactose and arabinose exceeds 70%. An arabinogalactan from Angelica sinensis (galactose 52.4%, arabinose 19.3%) significantly increases glutathione (GSH) levels and SOD activity while reducing MDA content [93]. Another from Taraxacum officinale (galactose 52.9%, arabinose 25.9%) exhibits excellent radical scavenging ability (DPPH, ABTS, OH) and reducing power, protects against H_2_O_2_-induced oxidative damage in vitro, enhances SOD activity, and reduces MDA levels [98].

Pectins also possess notable antioxidant activity. A pectin from Lycium barbarum (60.5% galacturonic acid) alleviates paraquat-induced oxidative stress in N2 worms, modulating NO production, activities of SOD, catalase (CAT), and glutathione reductase (GR), GSH and GSSG levels, GSH/GSSG ratio, and MDA content [190]. A pectin (galacturonic acid content: 49.2%) from Bupleurum chinense demonstrates potent scavenging of DPPH, ABTS, OH, and superoxide radicals in vitro, increases total antioxidant capacity (T-AOC), SOD, and GSH-Px activities, and reduces MDA levels in H_2_O_2_-treated SH-SY5Y cells [192]. Another pectin from Morus alba (61.0% galacturonic acid) shows strong Fe^2+^ chelating ability and scavenging activity against DPPH, OH, SOD, and ABTS radicals [196].

**Figure 6:**
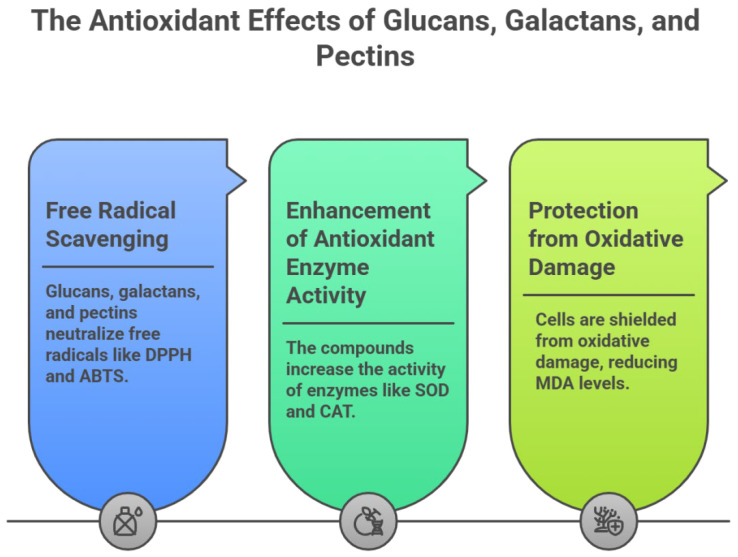
Mechanism of Antioxidant Action in Medicinal Plant Polysaccharides. Glucans, galactans, and pectins exhibit broad antioxidant effects through direct radical scavenging (1,1-diphenyl-2-picrylhydrazyl (DPPH) and 2,2′-azino-bis-(3-ethylbenzthiazoline-6-sulphonic acid) (ABTS)), reduction of oxidative stress (malondialdehyde (MDA)), and enhancement of intracellular antioxidant defence systems (serum superoxide dismutase (SOD) and catalase (CAT)).

In summary, glucans, galactans, and pectins each exhibit broad antioxidant effects through direct radical scavenging, reduction of oxidative stress, and enhancement of intracellular antioxidant defence systems. Their consistent mechanisms of action are illustrated in Figure 6. Furthermore, structural differences among these polysaccharides also influence their target tissues: the high galacturonic acid content and complex branching of pectins and arabinogalactans make them particularly effective in the liver and intestines [92,193], whereas glucans show distinctive potential in cellular protection and immune activation [48].

### 5.3. Regulation of Intestinal Flora

The gut, the largest organ in the human body, plays essential roles in digestion and immunity and harbours a complex microbial ecosystem. The gut microbiota is involved in numerous physiological processes, including nutrient absorption, metabolism, and immune regulation [223]. Given their notable capacity to modulate the gut microbiota, polysaccharides have garnered increasing research attention in recent years. As indicated in Table 2, fructans exhibit favourable effects on gut microbiota regulation. These polysaccharides possess a simple monosaccharide composition, typically containing over 90% fructose.

For example, a fructan from Ophiopogon japonicus (100% fructose) ameliorates microbial diversity and increases the relative abundance of beneficial bacteria, especially short-chain fatty acid (SCFA)-producing bacteria in high fat-diet (HFD)-induced obesity mice. Following microbial fermentation, it elevates levels of acetic and valeric acids, thereby regulating inflammatory responses and hepatic lipid metabolism [144]. Similarly, a fructan from Polygonati kingianum (91.3% fructose) enhanced the expression of tight junction proteins (zonula occludens-1 and occludin) and restored intestinal microbiota diversity by increasing the abundance of Firmicutes and reducing the abundance of Verrucomicrobiota [147]. Fructans from Polygonatum cyrtonema also showed prebiotic effects: one (77.4% fructose) protects the intestinal barrier, regulates SCFA levels, and promotes beneficial bacteria while inhibiting pathogens [150]; another (89.4% fructose) stimulates SCFA production and increases the abundance of beneficial bacteria such as Megamonas, Bifidobacterium, and Phascolarctobacterium, thereby changing microbial composition [152]. Noteworthy, previous research has proven that Bifidobacteria are considered key players in maintaining intestinal homeostasis [224,225].

Furthermore, extensive research has focused on the interactions between the gut microbiota, the gut, and the brain, commonly referred to as the “microbiota-gut-brain axis [226]”. Metabolites produced by the gut microbiota, particularly SCFAs, serve as key mediators in this bidirectional communication [227]. For example, a fructan (93.56% fructose) extracted from Polygonatum kingianum demonstrated significant neuro-regenerative activity in a mouse model of spinal cord injury by inhibiting excessive microglial activation, reducing neuroinflammation, and promoting neuronal survival and axonal regeneration [148]. However, fructans do not act directly on the brain or spinal cord but instead exert their effects by regulating the brain-gut axis. By reshaping the gut microbiota, they significantly increase the abundance of beneficial bacteria within the intestines, thereby elevating systemic circulation levels of their metabolic product, butyrate. Elevated butyrate acts as a key signalling molecule, ultimately exerting a remote inhibitory effect on inflammatory responses within the central nervous system.

**Figure 7:**
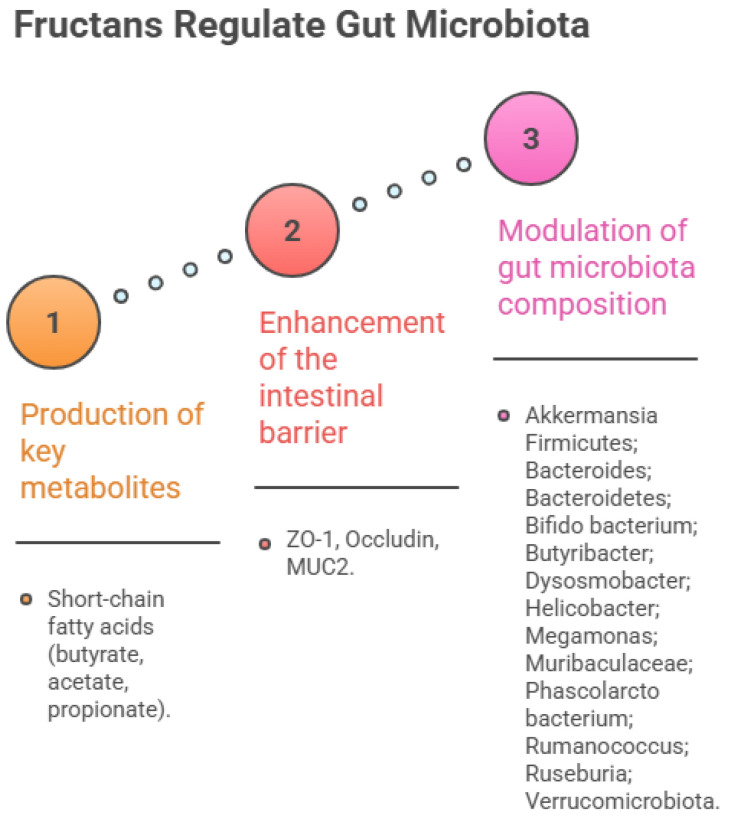
Mechanism of Gut Microbiota Regulation by Medicinal Plant Polysaccharides.

Fructans are the common name of a polysaccharide consisting of β-D-fructose. Although the small intestine does not produce any human digestive enzymes that can hydrolyse the β-glycosidic linkages, most gut microbiota can ferment fructans to promote the production of short-chain fatty acids (SCFAs), regulate the composition of the gut microbiota, maintain intestinal barrier integrity, and restore microbial metabolite levels [149,152]. The mechanism of action is shown in Figure 7. These properties support the potential development of fructans as prebiotic agents.

Fructans exert their overall regulation of the gut microbiota through enhancing intestinal barrier function (Zonula Occludens-1 (ZO-1), Occludin, Mucin 2 (MUC2)), producing key metabolites (short-chain fatty acids (butyrate, acetate, propionate)) and modulating the composition of the gut microbiota.

### 5.4. Other Activities

As summarised in Table 2, glucans display notable bioactivity in liver protection, neuroprotection, and tumour inhibition. Galactans have shown anti-ageing properties, mannans exhibit immunomodulatory potential, fructans combine immunomodulatory and antioxidant functions, and pectins hold promise for hypoglycaemic applications.

The biological activities of medicinal plant polysaccharides are closely linked to their highly complex and heterogeneous structures. Such structural diversity underlies their wide range of physiological effects. To date, however, the structure–activity relationships of these polysaccharides have not been elucidated, and the connections between specific structural features and their corresponding functions require further investigation.

## 6. Conclusions

Polysaccharides possess highly complex structural characteristics. These include molecular weight, monosaccharide composition, glycosidic bond configuration, and functional groups [4]. It is these structural features that determine and influence the biological activity of polysaccharides. Monosaccharides are the most fundamental units of the primary structure of polysaccharides and form the basis for more complex structures [5]. This review summarises high-quality literature from recent years concerning the influence of monosaccharide composition on the biological activity of polysaccharides.

The results are as follows: glucans are vital for immune regulation, antioxidant effects, and the regulation of gut microbiota. Similarly, galactans are important for antioxidant effects, immune regulation, and the regulation of gut microbiota. Mannans play a critical role in immune regulation, anti-tumour effects, and neuroprotection. Fructans play a critical role in regulating gut microbiota, immune regulation, and antioxidant effects. Pectins play a critical role in immune regulation, antioxidant effects, and lowering blood sugar.

Based on the fact that monosaccharide composition is a key factor affecting the activity of medicinal plant polysaccharides, subsequent researchers may consider isolating and purifying total polysaccharides to obtain target polysaccharides in order to improve their bioactivity. An in-depth and comprehensive understanding of the structure–activity relationship between the monosaccharide composition and biological activity of medicinal plant polysaccharides is expected to lead to the development and production of functional polysaccharides.

## Figures and Tables

**Table 1:** Monosaccharide composition and the bioactivity of medicinal plant polysaccharides.

No.	Source	Types of Polysaccharides	Glucose (%)	Galactose (%)	Arabinose (%)	Mannose (%)	Rhamnose (%)	Xylose (%)	Fucose (%)	Glucuronic Acid (%)	Galacturonic Acid (%)	Fructose (%)	Bioactivity	Reference
1	*Astragalus membranaceus*	Glucans	89.00	3.00	3.50	1.00	-	-	-	-	-	-	Immunomodulatory activity	[11]
2	*Astragalus membranaceus*	Glucans	95.76	1.83	2.41	-	-	-	-	-	-	-	Immunomodulatory activity	[12]
3	*Astragalus membranaceus*	Glucans	100	-	-	-	-	-	-	-	-	-	Immunomodulatory activity	[13]
4	*Astragalus membranaceus*	Glucans	91.69	4.18	4.13	-	-	-	-	-	-	-	Immunomodulatory activity	[14]
5	*Gastrodia elata*	Glucans	92.04	4.79	2.19	-	-	-	-	-	-	-	Immunomodulatory activity	[15]
6	*Sagittaria sagittifolia*	Glucans	91.76	7.72	0.27	-	-	-	-	-	-	-	Immunomodulatory activity	[16]
7	*Panax ginseng*	Glucans	100	-	-	-	-	-	-	-	-	-	Immunomodulatory activity	[17]
8	*Dendrobium huoshanense*	Glucans	95.46	-	-	4.54	-	-	-	-	-	-	Immunomodulatory activity	[18]
9	*Dendrobium officinale*	Glucans	94.17	-	-		-	-	-	5.82	-	-	Immunomodulatory activity	[19]
10	*Angelica dahurica*	Glucans	97.50	0.41	-	0.82	-	1.15	-	-	-	-	Immunomodulatory activity	[20]
11	*Angelica sinensis*	Glucans	97.83	-	-	1.19	-	-	-	-	-	-	Immunomodulatory activity	[21]
12	*Polygonum multiflorum*	Glucans	100.00	-	-	-	-	-	-	-	-	-	Immunomodulatory activity	[22]
13	*Radix Aconiti Lateralis Preparata*	Glucans	92.50	-	7.50	-	-	-	-	-	-	-	Immunomodulatory activity	[23]
14	*Schisandra chinensis*	Glucans	71.43	22.28	-	6.27	-	-	-	-	-	-	Immunomodulatory activity	[24]
15	*Schisandra chinensis*	Glucans	89.80	10.20	-	-	-	-	-	-	-	-	Immunomodulatory activity	[25]
16	*Gastrodia elata*	Glucans	88.21	4.48	-	-	-	-	-	-	4.40	-	Immunomodulatory activity	[26]
17	*Pueraria lobata*	Glucans	95.74	2.19	1.25	0.30	-	0.43	0.09	-	-	-	Immunomodulatory activity	[27]
18	*Pueraria lobata*	Glucans	100.00	-	-	-	-	-	-	-	-	-	Immunomodulatory activity	[28]
19	*Glehnia littorali*	Glucans	100.00	-	-	-	-	-	-	-	-	-	Immunomodulatory activity	[29]
20	*Astragalus membranaceus*	Glucans	97.00	-	-	-	-	-	-	-	-	-	Regulation of intestinal flora	[30]
21	*Astragalus membranaceus*	Glucans	83.01	1.22	15.40	-	-	-	-	-	-	-	Regulation of intestinal flora	[31]
22	*Astragalus membranaceus*	Glucans	76.34	-	17.51	-	-	-	-	-	-	-	Regulation of intestinal flora	[32]
23	*Astragalus membranaceus*	Glucans	89.78	4.30	4.39	1.53	-	-	-	-	-	-	Regulation of intestinal flora	[33]
24	*Panax ginseng*	Glucans	95.30	3.30	1.40	-	-	-	-	-	-	-	Regulation of intestinal flora	[34]
25	*Crataegus pinnatifida*	Glucans	95.37	0.42	0.79	0.15	0.70	-	-	-	2.34	-	Regulation of intestinal flora	[35]
26	*Lycium barbarum*	Glucans	98.10	-	-	-	-	-	-	-	-	-	Regulation of intestinal flora	[36]
27	*Atractylodis macrocephalae*	Glucans	84.16	6.51	9.33	-	-	-	-	-	-	-	Regulation of intestinal flora	[37]
28	*Lycium barbarum*	Glucans	81.83	2.02	3.46	6.52	6.06	-	-	-	-	-	Neuroprotective activity	[38]
29	*Schisandra chinensis*	Glucans	87.00	13.00	-	-	-	-	-	-	-	-	Neuroprotective activity	[39]
30	*Gastrodia elata*	Glucans	99.10	0.90	-	-	-	-	-	-	-	-	Neuroprotective activity	[40]
31	*Gastrodia elata*	Glucans	97.90	-	2.10	-	-	-	-	-	-	-	Neuroprotective activity	[41]
32	*Gastrodia elata*	Glucans	100.00	-	-	-	-	-	-	-	-	-	Neuroprotective activity	[42]
33	*Lonicera japonica*	Glucans	100.00	-	-	-	-	-	-	-	-	-	Neuroprotective activity	[43]
34	*Corydalis yanhusuo*	Glucans	100.00	-	-	-	-	-	-	-	-	-	Neuroprotective activity	[44]
35	*Dendrobium officinale*	Glucans	77.87	-	-	22.12	-	-	-	-	-	-	Antioxidant activity	[45]
36	*Angelica sinensis*	Glucans	76.34	14.81	2.63	5.78	-	-	-	-	-	-	Antioxidant activity	[46]
37	*Polygonum multiflorum*	Glucans	100.00	-	-	-	-	-	-	-	-	-	Antioxidant activity	[47]
38	*Glycyrrhiza inflata*	Glucans	79.08	10.50	10.42	-	-	-	-	-	-	-	Antioxidant activity	[48]
39	*Glycyrrhiza glabra*	Glucans	98.03	-	-	-	-	-	-	-	-	-	Antioxidant activity	[49]
40	*Pouteria campechiana*	Glucans	86.65	-	-	4.62	-	-	-	-	-	-	Antioxidant activity	[50]
41	*Taraxacum officinale*	Glucans	79.30	10.00	8.80	-	1.50	-	-	-	-	-	Antioxidant activity	[51]
42	*Sophora flavescens*	Glucans	78.75	9.17	8.34	2.49	0.30	0.95	-	-	-	-	Antioxidant activity	[52]
43	*Fallopia multiflora*	Glucans	100.00	-	-	-	-	-	-	-	-	-	Antioxidant activity	[53]
44	*Astragalus membranaceus*	Glucans	97.51	1.56	0.93	-	-	-	-	-	-	-	Hypoglycaemic activity	[54]
45	*Angelica sinensis*	Glucans	84.59	8.90	-	-	6.36	-	-	-	-	-	Hypoglycaemic activity	[55]
46	*Codonopsis Pilosula*	Glucans	71.38	24.98	3.60	-	-	-	-	-	-	-	Hypoglycaemic activity	[56]
47	*Glycyrrhiza uralensis*	Glucans	78.38	7.51	5.55	2.82	0.65	3.96	0.65	-	0.48	-	Hypoglycaemic activity	[57]
48	*Lycium barbarum*	Glucans	81.83	2.02	3.46	6.52	6.06	-	-	-	-	-	Hepatoprotective activity	[58]
49	*Polygonatum sibiricum*	Glucans	98.10	-	-	-	-	-	-	-	-	-	Hepatoprotective activity	[59]
50	*Schisandra chinensis*	Glucans	77.80	4.10	7.74	-	-	-	-	-	8.98	-	Hepatoprotective activity	[60]
51	*Puerariae lobatae*	Glucans	100.00	-	-	-	-	-	-	-	-	-	Hepatoprotective activity	[61]
52	*Puerariae thomsonii*	Glucans	100.00	-	-	-	-	-	-	-	-	-	Hepatoprotective activity	[62]
53	*Puerariae thomsonii*	Glucans	100.00	-	-	-	-	-	-	-	-	-	Hepatoprotective activity	[63]
54	*Cyathulae officinalis*	Glucans	93.34	6.65	-	-	-	-	-	-	-	-	Hepatoprotective activity	[64]
55	*Ginkgo biloba*	Glucans	98.12	1.10	0.80	-	-	-	-	-	-	-	Hepatoprotective activity	[65]
56	*Dendrobium officinale*	Glucans	68.15	-	-	31.85	-	-	-	-	-	-	Antitumor activity	[66]
57	*Angelica sinensis*	Glucans	93.15	-	6.75	-	-	-	-	-	-	-	Antitumor activity	[67]
58	*Platycodon grandiflorus*	Glucans	92.80	2.85	1.11	0.26	-	-	-	1.14	1.83	-	Antitumor activity	[68]
59	*Atractylodes macrocephala*	Glucans	82.10	-	17.90	-	-	-	-	-	-	-	Antitumor activity	[69]
60	*Glehnia littoralis*	Glucans	92.10	5.30	2.60	-	-	-	-	-	-	-	Antitumor activity	[70]
61	*Pseudostellaria heterophylla*	Glucans	93.10	1.00	0.90	-	-	-	-	2.50	0.50	-	Antitumor activity	[71]
62	*Angelica pubescens*	Glucans	85.10	4.50	3.20	7.30	-	-	-	-	-	-	Anti-inflammatory activity	[72]
63	*Dioscorea opposita*	Glucans	79.72	3.03	1.45	14.90	0.22	0.42	-	-	-	-	Anti-inflammatory activity	[73]
64	*Gastrodia elata*	Glucans	66.12	-	-	-	-	-	-	-	-	31.76	Anti-inflammatory activity	[74]
65	*Gastrodia elata*	Glucans	89.69	-	-	-	-	-	-	-	-	10.31	Anti-inflammatory activity	[75]
66	*Lycium ruthenicum*	Arabinogalactans	-	39.52	56.62	-	3.80	-	-	-	-	-	Immunomodulatory activity	[76]
67	*Lycium barbarum*	Arabinogalactans	-	31.07	63.79	-	1.23	-	-	-	3.89	-	Immunomodulatory activity	[77]
68	*Scutellaria baicalensis*	Arabinogalactans	-	22.20	67.10	-	4.40	-	-	1.20	6.30	-	Immunomodulatory activity	[78]
69	*Rehmannia glutinosa*	Arabinogalactans	0.05	56.60	38.10	-	-	-	-	-	-	-	Immunomodulatory activity	[79]
70	*Atractylodes lancea*	Arabinogalactans	-	35.00	50.00	-	14.50	4.00	-	-	-	-	Immunomodulatory activity	[80]
71	*Astragalus membranaceus*	Arabinogalactans	6.34	27.39	48.39	1.61	6.05	-	-	-	10.21	-	Immunomodulatory activity	[81]
72	*Astragalus membranaceus*	Arabinogalactans	13.77	18.36	51.00	-	1.53	-	-	-	15.30	-	Immunomodulatory activity	[82]
73	*Atractylodes lancea*	Arabinogalactans	3.01	11.21	70.82	-	8.84	1.84	-	-	4.28	-	Immunomodulatory activity	[83]
74	*Dendrobium officinale*	Arabinogalactans	-	46.79	29.79	-	11.68	-	-	-	11.80	-	Regulation of intestinal flora	[84]
75	*Lycium barbarum*	Arabinogalactans	-	45.00	55.00	-	-	-	-	-	-	-	Regulation of intestinal flora	[85]
76	*Lycium barbarum*	Arabinogalactans	12.40	21.92	38.52	-	15.88	-	-	-	10.47	-	Regulation of intestinal flora	[86]
77	*Lycium barbarum*	Arabinogalactans	10.22	30.2	48.18	-	5.23	-	-	-	2.57	-	Regulation of intestinal flora	[87]
78	*Lycium barbarum*	Arabinogalactans	2.15	39.67	40.66	-	-	-	-	5.12	12.40	-	Regulation of intestinal flora	[88]
79	*Angelica sinensis*	Arabinogalactans	-	62.08	30.36	-	-	-	-	-	7.57	-	Regulation of intestinal flora	[89]
80	*Atractylodes chinensis*	Arabinogalactans	-	44.10	55.90	-	-	-	-	-	-	-	Regulation of intestinal flora	[90]
81	*Angelica sinensis*	Arabinogalactans	17.75	52.41	19.31	-	-	-	-	10.44	-	-	Antioxidant activity	[91]
82	*Angelica sinensis*	Arabinogalactans	17.75	52.40	19.31	-	-	-	-	10.44	-	-	Antioxidant activity	[92]
83	*Angelica sinensis*	Arabinogalactans	17.75	52.40	19.31	-	-	-	-	10.44	-	-	Antioxidant activity	[93]
84	*Bupleurum chinense*	Arabinogalactans	17.80	44.50	37.38	-	-	-	-	-	-	-	Antioxidant activity	[94]
85	*Zizyphus Jujuba*	Arabinogalactans	3.41	55.40	33.30	2.44	4.06	-	-	-	1.42	-	Antioxidant activity	[95]
86	*Pueraria mirifica*	Arabinogalactans	4.50	58.50	27.80	0.60	7.40	-	-	0.80	0.20	-	Antioxidant activity	[96]
87	*Taraxacum officinale*	Arabinogalactans	9.43	42.24	43.84	2.35	2.07	-	-	-	-	-	Antioxidant activity	[97]
88	*Taraxacum officinale*	Arabinogalactans	8.07	52.94	25.95	7.33	1.84	-	-	1.47	2.40	-	Antioxidant activity	[98]
89	*Ginkgo biloba*	Arabinogalactans	5.94	54.00	17.28	4.32	6.48	-	-	8.64	3.24	-	Antioxidant activity	[99]
90	*Panax ginseng*	Arabinogalactans	-	22.40	53.80	-	10.30	-	-	-	13.20	-	Antioxidant activity	[100]
91	*Polygonatum sibiricum*	Arabinogalactans	-	73.64	21.04	-	5.26	-	-	-	-	-	Antioxidant activity	[101]
92	*Lycium barbarum*	Arabinogalactans	18.84	30.22	43.09	-	-	-	-	-	-	-	Anti-ageing activity	[102]
93	*Lycium barbarum*	Arabinogalactans	-	45.90	46.10	-	-	-	-	-	-	-	Anti-ageing activity	[103]
94	*Rehmannia glutinosa*	Arabinogalactans	6.68	37.83	55.49	-	-	-	-	-	-	-	Anti-ageing activity	[104]
95	*Rehmannia glutinosa*	Arabinogalactans	15.39	61.36	18.19	0.80	3.31	-	-	-	0.96	-	Anti-ageing activity	[105]
96	*Lycium barbarum*	Arabinogalactans	-	60.93	39.06	-	-	-	-	-	-	-	Neuroprotective activity	[106]
97	*Lycium barbarum*	Arabinogalactans	6.89	37.64	34.88	1.03	3.68	2.46	-	0.73	12.67	-	Neuroprotective activity	[107]
98	*Lycium barbarum*	Arabinogalactans	1.40	49.80	47.80	-	1.20	-	-	-	-	-	Neuroprotective activity	[108]
99	*Ginkgo biloba*	Arabinogalactans	3.00	5.00	82.00	5.00	-	-	-	-	-	-	Neuroprotective activity	[109]
100	*Lycium barbarum*	Arabinogalactans	14.72	28.08	37.53	4.50	-	7.83	-	-	-	-	Antitumor activity	[110]
101	*Lycium ruthenicum*	Arabinogalactans	16.83	29.35	43.51	1.75	3.29	-	-	2.11	3.16	-	Antitumor activity	[111]
102	*Angelica sinensis*	Arabinogalactans	17.75	52.41	19.31	-	-	-	-	10.44	-	-	Antitumor activity	[112]
103	*Panax notoginseng*	Arabinogalactans	-	43.70	56.30	-	-	-	-	-	-	-	Antitumor activity	[113]
104	*Ophiopogon japonicus*	Galactans	-	100.00	-	-	-	-	-	-	-	-	Antitumor activity	[114]
105	*Polygonatum cyrtonema*	Galactans	-	100.00	-	-	-	-	-	-	-	-	Regulation of intestinal flora	[115]
106	*Polygonatum cyrtonema*	Galactans	-	100.00	-	-	-	-	-	-	-	-	Regulation of intestinal flora	[116]
107	*Polygonatum sibiricum*	Galactans	2.13	82.91	-	14.96	-	-	-	-	-	-	Immunomodulatory activity	[117]
108	*Polygonatum sibiricum*	Galactans	-	78.77	-	5.50	-	-	-	-	13.84	-	Immunomodulatory activity	[118]
109	*Rehmannia glutinosa*	Arabinans	-	-	100.00	-	-	-	-	-	-	-	Immunomodulatory activity	[119]
110	*Glehnia littoralis*	Arabinans	-	-	100.00	-	-	-	-	-	-	-	Antitumor activity	[120]
111	*Akebia quinata*	Arabinans	-	-	100.00	-	-	-	-	-	-	-	Immunomodulatory activity	[121]
112	*Dendrobium officinale*	Glucomannans	33.3	16.60	-	50.00	-	-	-	-	-	-	Immunomodulatory activity	[122]
113	*Dendrobium officinale*	Glucomannans	17.97	-	-	82.03	-	-	-	-	-	-	Immunomodulatory activity	[123]
114	*Dendrobium officinale*	Glucomannans	28.36	-	-	70.43	-	-	-	-	-	-	Immunomodulatory activity	[124]
115	*Dendrobium officinale*	Glucomannans	14.80	-	-	85.20	-	-	-	-	-	-	Immunomodulatory activity	[125]
116	*Anemarrhena asphodeloides*	Glucomannans	10.90	2.60	7.30	79.00	-	0.20	-	-	-	-	Immunomodulatory activity	[126]
117	*Dendrobium wardianum*	Glucomannans	22.85	-	-	76.66	-	-	-	-	-	-	Antitumor activity	[127]
118	*Dendrobium officinale*	Glucomannans	22.84	-	-	77.16	-	-	-	-	-	-	Antitumor activity	[128]
119	*Platycodon grandiflorum*	Glucomannans	42.00	-	-	57.96	-	-	-	-	-	-	Antitumor activity	[129]
120	*Dendrobium officinale*	Glucomannans	17.92	-	-	82.08	-	-	-	-	-	-	Neuroprotective activity	[130]
121	*Dendrobium huoshanense*	Glucomannans	24.19	-	-	75.81	-	-	-	-	-	-	Neuroprotective activity	[131]
122	*Dendrobium huoshanense*	Glucomannans	33.47	0.48	0.26	65.79	-	-	-	-	-	-	Gastroprotective activity	[132]
123	*Dendrobium huoshanense*	Glucomannans	24.75	-	-	75.25	-	-	-	-	-	-	Gastroprotective activity	[133]
124	*Dendrobium officinale*	Glucomannans	24.00	-	-	76.00	-	-	-	-	-	-	Hepatoprotective activity	[134]
125	*Dendrobium officinale*	Glucomannans	17.24	-	-	82.76	-	-	-	-	-	-	Renal protective activity	[135]
126	*Dendrobium officinale*	Glucomannans	28.17	-	-	71.83	-	-	-	-	-	-	Regulation of intestinal flora	[136]
127	*Dendrobium huoshanense*	Glucomannans	36.07	1.65	-	62.25	-	-	-	-	-	-	Anti-osteoporosis activity	[137]
128	*Bletilla striata*	Glucomannans	25.00	-	-	75.00	-	-	-	-	-	-	Antioxidant activity	[138]
129	*Dendrobium officinale*	Mannans	5.09	2.29	1.46	91.15	-	-	-	-	-	-	Immunomodulatory activity	[139]
130	*Ginkgo biloba*	Mannans	-	2.91	-	97.08	-	-	-	-	-	-	Antioxidant activity	[140]
131	*Codonopsis pilosula*	Fructans	3.40	-	-	-	-	-	-	-	-	96.60	Regulation of intestinal flora	[141]
132	*Codonopsis pilosula*	Fructans	2.72	-	-	-	-	-	-	-	-	97.28	Regulation of intestinal flora	[142]
133	*Ophiopogon japonicus*	Fructans	-	-	-	-	-	-	-	-	-	100.00	Regulation of intestinal flora	[143]
134	*Ophiopogon japonicus*	Fructans	-	-	-	-	-	-	-	-	-	100.00	Regulation of intestinal flora	[144]
135	*Ophiopogon japonicus*	Fructans	-	-	-	-	-	-	-	-	-	100.00	Regulation of intestinal flora	[145]
136	*Ophiopogon japonicus*	Fructans	-	-	-	-	-	-	-	-	-	100.00	Regulation of intestinal flora	[146]
137	*Polygonati kingianum*	Fructans	6.90	-	-	0.90	-	-	-	-	-	91.30	Regulation of intestinal flora	[147]
138	*Polygonatum kingianum*	Fructans	6.44	-	-	-	-	-	-	-	-	93.56	Regulation of intestinal flora	[148]
139	*Polygonatum cyrtonema*	Fructans	3.44	-	-	-	-	-	-	-	-	96.32	Regulation of intestinal flora	[149]
140	*Polygonatum cyrtonema*	Fructans	7.50	-	-	7.40	-	-	-	-	-	77.40	Regulation of intestinal flora	[150]
141	*Polygonatum kingianum*	Fructans	7.10	-	-	-	-	-	-	-	-	92.90	Regulation of intestinal flora	[151]
142	*Polygonatum cyrtonema*	Fructans	5.84	-	-	3.18	-	-	-	-	-	89.48	Regulation of intestinal flora	[152]
143	*Atractylodes lancea*	Fructans	5.52	-	-	-	-	-	-	-	-	94.48	Regulation of intestinal flora	[153]
144	*Codonopsis pilosula*	Fructans	-	-	-	-	-	-	-	-	-	100.00	Immunomodulatory activity	[154]
145	*Polygonatum cyrtonema*	Fructans	19.19	-	-	-	-	-	-	-	-	80.81	Immunomodulatory activity	[155]
146	*Polygonatum odoratum*	Fructans	3.30	-	-	-	-	-	-	-	-	96.70	Immunomodulatory activity	[156]
147	*Atractylodis Macrocephalae*	Fructans	11.00	-	-	-	-	-	-	-	-	89.00	Immunomodulatory activity	[157]
148	*Anemarrhena asphodeloides*	Fructans	5.50	-	-	-	-	-	-	-	-	94.50	Immunomodulatory activity	[158]
149	*Polygonatum cyrtonema*	Fructans	6.37	0.90	-	-	-	-	-	-	-	92.73	Antioxidant activity	[159]
150	*Polygonatum sibiricum*	Fructans	5.40	-	-	3.60	-	-	-	-	-	91.00	Antioxidant activity	[160]
151	*Polygonatum cyrtonema*	Fructans	3.85	-	-	-	-	-	-	-	-	95.89	Antioxidant activity	[161]
152	*Polygonatum kingianum*	Fructans	7.20	0.80	-	-	-	-	-	-	-	92.00	Antioxidant activity	[162]
153	*Liriope spicata*	Fructans	3.33	-	-	-	-	-	-	-	-	96.57	Hypoglycaemic activity	[163]
154	*Ophiopogon japonicas*	Fructans	-	-	-	-	-	-	-	-	-	100.00	Hypoglycaemic activity	[164]
155	*Polygonatum kingianum*	Fructans	-	11.20	-	1.10	-	-	-	-	-	87.70	Hypoglycaemic activity	[165]
156	*Codonopsis pilosula*	Fructans	3.17	-	2.40	-	-	-	-	-	-	94.21	Hepatoprotective activity	[166]
157	*Ophiopogon japonicus*	Fructans	3.13	-	-	-	-	-	-	-	-	96.86	Hepatoprotective activity	[167]
158	*Plantago asiatica*	Araboxylans	-	-	32.20	-	-	61.10	-	-	-	-	Regulation of intestinal flora	[168]
159	*Plantago asiatica*	Araboxylans	-	-	32.20	-	-	61.10	-	-	-	-	Hypoglycaemic activity	[169]
160	*Prunella vulgaris*	Araboxylans	8.30	9.70	24.20	1.90	-	55.90	-	-	-	-	Immunomodulatory activity	[170]
161	*Panax ginseng*	Pectins	18.20	19.40	7.90	-	5.20	-	-	-	49.30	-	Immunomodulatory activity	[171]
162	*Panax ginseng*	Pectins	1.90	41.20	7.30	0.80	-	-	-	-	45.80	-	Immunomodulatory activity	[172]
163	*Panax ginseng*	Pectins	2.00	5.90	-	-	-	-	-	-	92.10	-	Immunomodulatory activity	[173]
164	*Panax ginseng*	Pectins	3.00	19.50	9.20	0.40	21.80	-	-	2.20	33.80	-	Immunomodulatory activity	[174]
165	*Panax ginseng*	Pectins	12.28	14.58	15.53	-	9.86	-	-	-	47.74	-	Immunomodulatory activity	[175]
166	*Panax ginseng*	Pectins	3.00	19.50	9.20	-	21.80	-	-	-	33.80	-	Immunomodulatory activity	[176]
167	*Codonopsis pilosula*	Pectins	-	-	3.50	-	5.70	-	-	-	90.80	-	Immunomodulatory activity	[177]
168	*Codonopsis pilosula*	Pectins	-	4.92	2.92	-	7.59	-	-	-	84.55	-	Immunomodulatory activity	[178]
169	*Angelica sinensis*	Pectins	4.30	21.60	22.40	7.50	3.50	-	-	-	39.00	-	Immunomodulatory activity	[179]
170	*Panax notoginseng*	Pectins	4.50	33.30	25.20	-	15.50	-	-	-	17.10	-	Immunomodulatory activity	[180]
171	*Plantago asiatica*	Pectins	5.67	24.00	15.89	3.79	17.89	7.12	1.11	1.86	22.68	-	Immunomodulatory activity	[181]
172	*Panax quinquefolius*	Pectins	11.50	15.20	19.20	12.00	2.10	9.60	-	4.10	26.30	-	Immunomodulatory activity	[182]
173	*Pueraria lobata*	Pectins	4.05	16.60	16.52	0.48	6.14	4.75	2.54	1.47	47.44	-	Immunomodulatory activity	[183]
174	*Atractylodis Macrocephalae*	Pectins	-	4.20	6.80	-	11.00	-	-	-	77.90	-	Immunomodulatory activity	[184]
175	*Gardenia jasminoides*	Pectins	6.03	18.52	20.30	-	5.02	-	-	-	50.14	-	Immunomodulatory activity	[185]
176	*Ginkgo biloba*	Pectins	1.97	6.00	7.86	0.44	6.95	0.57	0.61	2.43	73.18	-	Immunomodulatory activity	[186]
177	*Saposhnikovia divaricata*	Pectins	-	5.80	7.60	-	1.60	-	-	-	85.60	-	Immunomodulatory activity	[187]
178	*Saposhnikovia divaricata*	Pectins	-	43.00	35.00	-	2.00	-	-	-	20.00	-	Immunomodulatory activity	[188]
179	*Lycium barbarum*	Pectins	6.15	17.15	4.16	-	18.50	3.36	-	-	46.91	-	Antioxidant activity	[189]
180	*Lycium barbarum*	Pectins	7.37	9.95	8.93	2.47	7.00	1.16	-	-	60.55	-	Antioxidant activity	[190]
181	*Codonopsis pilosula*	Pectins	-	11.00	8.90	-	9.30	-	-	-	70.10	-	Antioxidant activity	[191]
182	*Bupleurum chinense*	Pectins	2.50	16.70	12.90	-	14.20	1.60	-	-	49.20	-	Antioxidant activity	[192]
183	*Salvia miltiorrhiza*	Pectins	4.00	5.60	5.60	-	5.20	-	-	-	78.80	-	Antioxidant activity	[193]
184	*Ziziphus jujuba*	Pectins	5.91	21.69	25.89	-	10.69	-	-	-	33.49	-	Antioxidant activity	[194]
185	*Polygonatum odoratum*	Pectins	-	10.90	6.10	-	4.40	1.10	-	1.00	76.50	-	Antioxidant activity	[195]
186	*Morus alba*	Pectins	-	12.70	8.90	-	15.70	-	-	2.00	61.00	-	Antioxidant activity	[196]
187	*Sophorae Tonkinensis*	Pectins	1.20	9.70	7.30	2.00	18.40	0.90	-	-	60.40	-	Antioxidant activity	[197]
188	*Codonopsis pilosula*	Pectins	-	4.16	4.16	-	8.32	-	-	-	83.20	-	Antitumor activity	[198]
189	*Bupieurum chinense*	Pectins	-	4.42	11.51	-	7.18	-	-	-	76.89	-	Antitumor activity	[199]
190	*Lycium barbarum*	Pectins	15.47	14.67	27.95	4.10	3.19	-	-	-	34.62	-	Antitumor activity	[200]
191	*Lycium ruthenicum*	Pectins	-	26.60	24.90	-	14.40	16.40	-	-	17.70	-	Antitumor activity	[201]
192	*Polygonum multiflorum*	Pectins	-	29.60	24.60	-	26.40	-	-	-	20.00	-	Antitumor activity	[202]
193	*Polygala tenuifolia*	Pectins	-	18.90	65.60	-	7.30	-	-	-	8.20	-	Antitumor activity	[203]
194	*Panax ginseng*	Pectins	18.50	18.00	15.50	-	2.50	-	-	-	44.20	-	Hypoglycaemic activity	[204]
195	*Lycium barbarum*	Pectins	5.29	19.53	23.10	3.49	2.77	3.46	-	-	42.33	-	Hypoglycaemic activity	[205]
196	*Lycium barbarum*	Pectins	-	3.09	37.29	14.30	4.75	1.76	-	-	38.76	-	Hypoglycaemic activity	[206]
197	*Ziziphus jujuba*	Pectins	4.05	9.48	3.29	-	9.13	-	-	-	68.71	-	Hypoglycaemic activity	[207]
198	*Schisandra chinensis*	Pectins	1.10	1.29	0.89	0.71	0.88	-	-	2.56	90.06	-	Hypoglycaemic activity	[208]
199	*Pseudostellaria heterophylla*	Pectins	-	7.00	20.50	-	5.10	-	-	-	63.20	-	Hypoglycaemic activity	[209]
200	*Lycium barbarum*	Pectins	-	8.78	15.41	-	-	-	-	-	75.81	-	Regulation of intestinal flora	[210]
201	*Gardenia jasminoides*	Pectins	3.18	4.16	4.72	-	5.91	-	-	-	82.03	-	Regulation of intestinal flora	[211]
202	*Lycium barbarum*	Pectins	3.90	20.42	43.84	0.97	6.20	-	-	-	24.67	-	Regulation of intestinal flora	[212]
203	*Morus alba*	Pectins	5.74	17.28	24.13	-	23.00	1.12	-	4.12	24.60	-	Regulation of intestinal flora	[213]
204	*Dendrobium nobile*	Pectins	0.43	3.55	4.47	-	2.38	17.84	0.26	1.26	69.80	-	Hepatoprotective activity	[214]
205	*Panax notoginseng*	Pectins	1.60	3.00	4.50	0.20	3.80	-	-	-	86.80	-	Hepatoprotective activity	[215]
206	*Crataegus pinnatifida*	Pectins	-	-	-	-	-	-	-	-	100.00	-	Hepatoprotective activity	[216]
207	*Gardenia jasminoides*	Pectins	-	3.22	4.14	-	5.77	-	-	-	86.87	-	Hepatoprotective activity	[217]
208	*scrophularia ningpoensis*	Pectins	-	24.00	13.50	5.40	1.20	0.50	-	4.40	51.10	-	Neuroprotective activity	[218]
209	*Polygala tenuifolia*	Pectins	-	19.80	63.50	-	8.30	-	-	-	8.40	-	Neuroprotective activity	[219]
210	*Eucommia ulmoides*	Pectins	1.54	9.43	34.22	0.97	18.32	-	0.14	1.27	34.11	-	Anti-osteoporosis activity	[220]

Note: No. indicates the serial number. Source indicates the plant’s Latin name.

**Table 2:** Summary of the Effects of Different Polysaccharide Types on Biological Activity.

Types of Polysaccharides	Immunomodulatory Activity	Antioxidant Activity	Regulation of Intestinal Flora	Hepatoprotective Activity	Neuroprotective Activity	Antitumor Activity	Hypoglycaemic Activity	Anti-Inflammatory Activity	Anti-Ageing Activity	Gastroprotective Activity	Renal Protective Activity	Anti-Osteoporosis Activity
Glucans	21	9	8	8	7	6	4	4	0	0	0	0
Galactans	9	11	7	0	4	4	0	0	4	0	0	0
Mannans	5	1	1	1	2	3	0	0	0	2	1	1
Fructans	5	4	14	2	0	0	3	0	0	0	0	0
Pectins	21	10	4	4	2	6	6	0	0	0	0	1

Note: The numbers in the table represent the number of literature references collected in this paper for biological activities of different polysaccharides.

## Data Availability

No new data were created or analysed in this study. Data sharing is not applicable to this article.
